# Nonketotic Hyperglycemic Chorea

**DOI:** 10.1155/2014/128037

**Published:** 2014-02-23

**Authors:** Mahmoud Abdelghany, Samuel Massoud

**Affiliations:** Department of Medicine, Conemaugh Memorial Medical Center, 1086 Franklin Street, E3 Building, Johnstown, PA 15905, USA

## Abstract

This is a unique case of nonketotic hyperglycemic (NKH) chorea in a 34-year-old white male. The patient had a poorly controlled type 2 diabetes mellitus (DM) due to medication incompliance. He complained of polyuria, polydipsia, and weight loss of 20 pounds within a month before presentation. T2-weighted (T2W) MRI showed hyperintensity in the left basal ganglion. Glycated hemoglobin (HBA1c) was 13.6%. The patient was started on insulin and clonazepam and the chorea resolved after proper control of the glucose level. To our knowledge, this is the first reported case of NKH chorea in a young white male with high T2-weighted (T2W) magnetic resonance signal in the basal ganglia.

## 1. Introduction

Chorea is an irregular, poorly patterned, involuntary movement disorder. Various conditions such as cerebrovascular insufficiency, neurodegenerative diseases, neoplastic diseases, immunological diseases, infectious diseases, and metabolic diseases are known as secondary causes of this rare disorder [1]. Hyperglycemia is the most common metabolic cause of chorea-ballism [2]. NKH chorea is more common in elderly patients [2–5] especially females from East Asian origin [3, 5]. Proper control of DM, with or without neuroleptic drugs, is the key for treatment.

## 2. Case

A 34-year-old white male presented to our hospital complaining of 1 month of flailing-like movements of his right upper extremity that progressed to the whole right side of the body and his neck. He also complained of a 20-pound weight loss within a month before presentation accompanied by polyuria and polydipsia. The patient denied any loss of consciousness, weakness, difficulty walking, headaches, blurry vision, slurred speech, fever, chills, or recent flu-like symptoms. He also denied taking any neuroleptic drugs. His past medical history was significant for type 2 DM for which he was on insulin but had not been taking it for a year because of financial problems.

**Figure 1:**
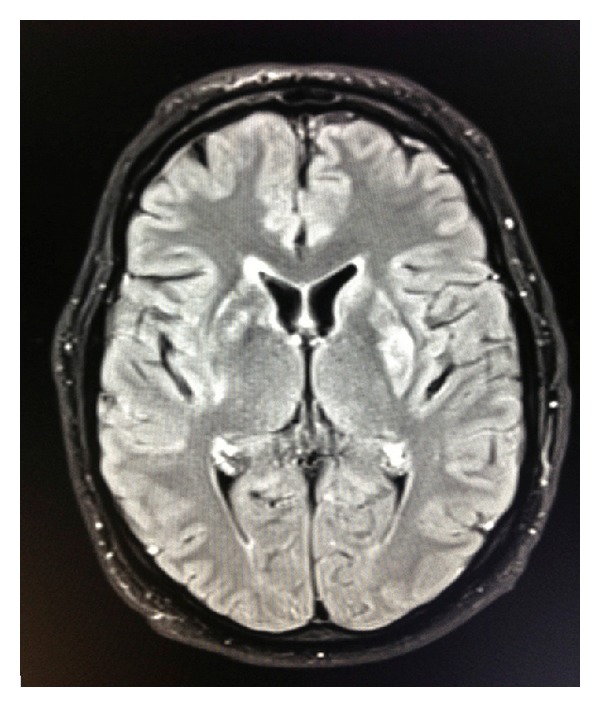
Axial Flair shows hyperintense signal in the right caudothalamic groove near the right foramen of Monro and in the left putamen.

**Figure 2:**
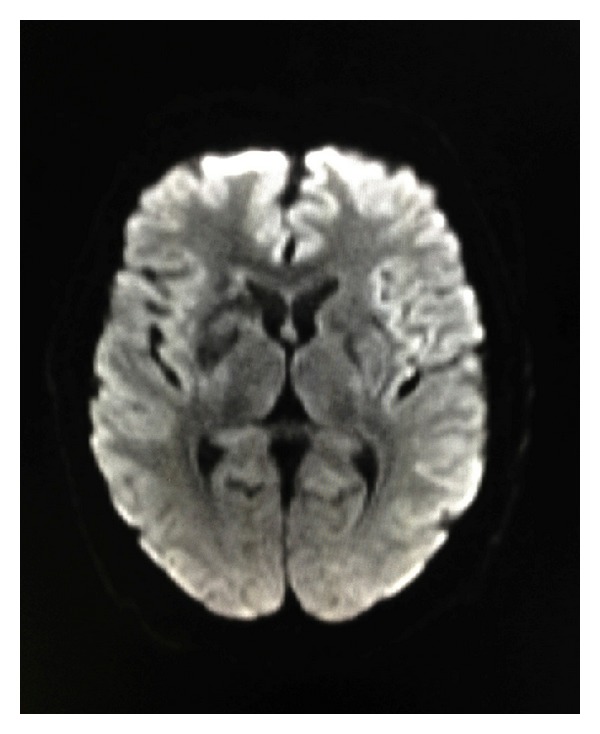
Axial DWI does not show evidence of restricted diffusion.

**Figure 3:**
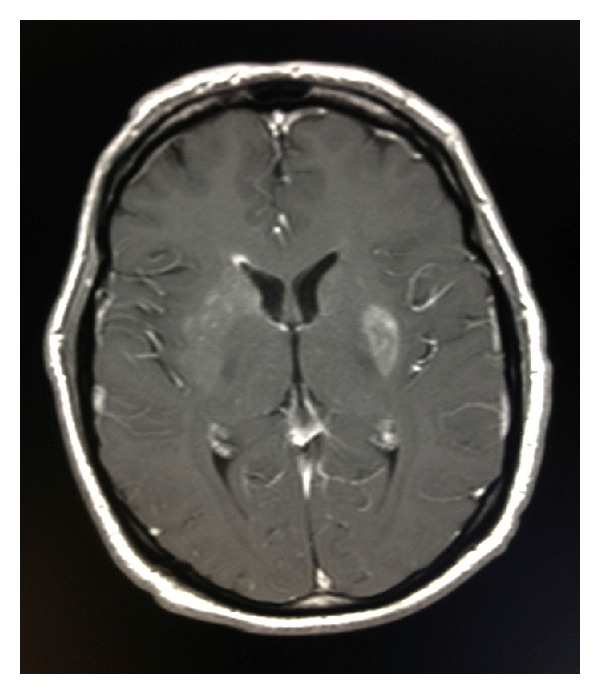
Axial T1 postgadolinium demonstrates enhancement along the periventricular white matter of the right frontal horn and in the left putamen.

**Figure 4:**
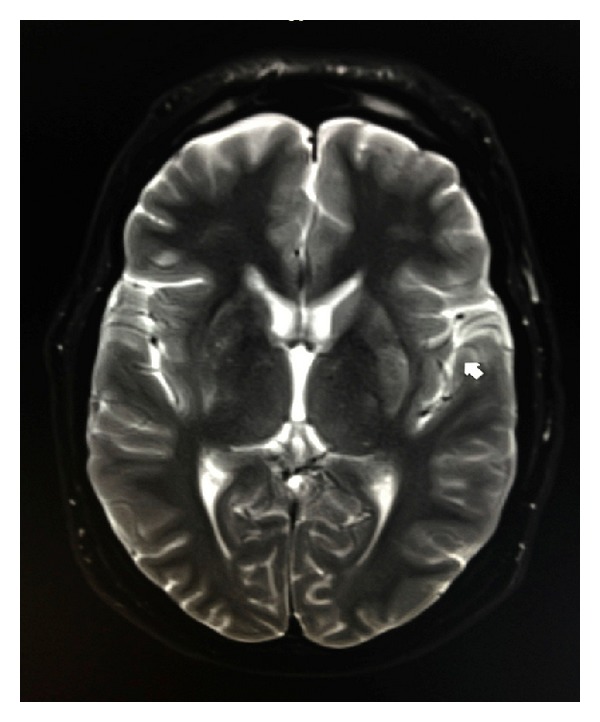
Axial T2 demonstrates hyperintensity within the left putamen.

**Figure 5:**
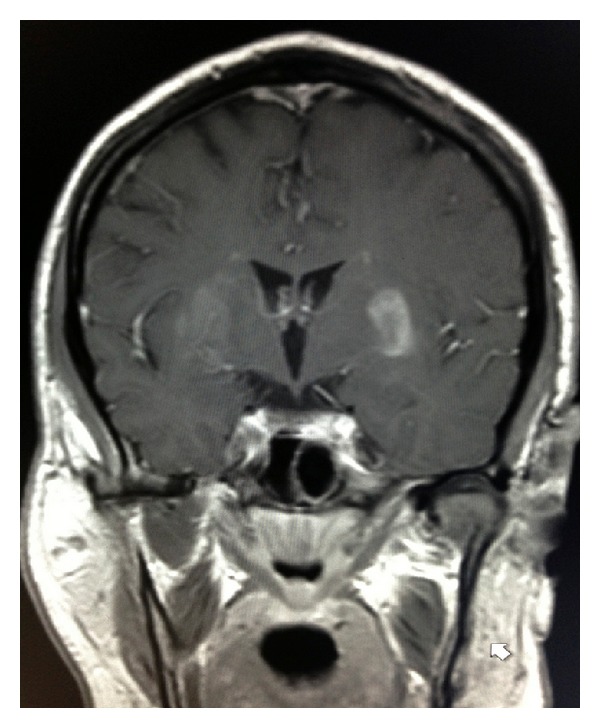
Coronal T1 postcontrast demonstrates enhancement in the left putamen and right caudothalamic groove.

Physical exam showed mild hypotonia in the right upper extremity with no weakness in all extremities. Laboratory investigations showed blood sugar around 230 mg/dL, with no anion gap. HBA1c was 13.6%. Urine drug screening was negative. CT scan of the head did not show any abnormalities. MRI of the brain (Figures 1, 2, 3, 4, and 5) showed high T2W signal in both putamina of the basal ganglia and low T1 weighted (T1W) signal in the left putamen of the basal ganglion with no restricted diffusion on the axial diffusion weighted imaging (DWI). Thyroid stimulating hormone, antinuclear antibody, antiphospholipid antibody, liver function tests, ceruloplasmin level, iron studies, angiotensin converting enzyme level, C-peptide, vitamins D and B12, and folic acid were either normal or negative.

The patient was started on insulin and clonazepam 0.5 mg daily. After 3 days, the patient's symptoms improved with marked decrease of the abnormal movement. Two weeks later, the abnormal movements disappeared completely. The patient refused to have a follow-up brain MRI after 6 months. One year later he continues to be asymptomatic with no abnormal movements.

## 3. Discussion

Chorea secondary to hyperglycemia was first reported in 1960 [6]. Since that time there have been similar cases reported worldwide; most of them are secondary to NKH in type 2 diabetic patients, although rare cases were reported in ketotic hyperglycemic type 1 diabetic patients [2]. The average age of onset is 71 years old [4], mostly in Asian females, which increases the possibilities of genetic or environmental predisposing factors [2].

Various hypotheses have been proposed to explain the pathophysiology of hyperglycemic chorea, but the exact mechanism is still unknown [2–5]. Cerebral vascular insufficiency, petechial hemorrhage [7], hyperviscosity [8], and depletion of both gamma-aminobutyric acid (GABA) and acetylcholine secondary to metabolic changes [9, 10] have been suggested as possible mechanisms of NKH chorea. Concurrent infection was reported in multiple cases [2, 3, 5] suggesting that this might play as a trigger factor for NKH chorea in predisposed patients. Acute putaminal dysfunction, secondary to hyperglycemic or hyperosmolar insult, associated with some degree of Wallerian degeneration of the internal white matter of the putamen has been also considered to play a pathogenic role in NKH chorea [11].

NKH chorea might be the first presentation of hyperglycemia [3–5, 12], or it might be secondary to poorly controlled DM. Some patients developed chorea after rapid correction of hyperglycemia [13]. Moreover, chorea can also occur in hypoglycemia [14]. Typically in patients with NKH chorea, there are high T1- and low T2-weighted signal in the contralateral putamen of the basal ganglia and restricted diffusion in the DWI. CT of the brain may show high density [15], but it might fail to show any abnormality in the basal ganglia by the time the MRI can detect it [2], as seen in our patient. Moreover, certain cases were reported having NKH chorea with absence of putamen abnormalities on the MRI of the brain [12].

Overall, the prognosis of NKH chorea has been reported to be excellent, with rare exception [12]. It depends on the prompt identification of undiagnosed diabetes or the proper control of the blood sugar in the previously diagnosed patients. Additionally, typical neuroleptic drugs and sometimes benzodiazepines are useful in the management of choreic movements [5]. A follow-up brain MRI after 6 months usually shows disappearance of the initial findings but they may persist for years. In our case, the patient's noncompliance with his medications and lack of medical care might be the reason he developed NKH chorea in this young age. To our knowledge, this is the first reported case of NKH chorea under the age of 35 with unusual finding of high T2W magnetic resonance signal.
